# Reproducibility of nighttime home blood pressure measured by a wrist‐type nocturnal home blood pressure monitoring device

**DOI:** 10.1111/jch.14342

**Published:** 2021-08-21

**Authors:** Naoko Tomitani, Hiroshi Kanegae, Kazuomi Kario

**Affiliations:** ^1^ Department of Medicine Division of Cardiovascular Medicine Jichi Medical University School of Medicine Tochigi Japan; ^2^ Genki Plaza Medical Center for Health Care Tokyo Japan

**Keywords:** nighttime blood pressure, reproducibility of nighttime home blood pressure, wrist‐type home nocturnal blood pressure monitor

## Abstract

The authors investigated the reproducibility of nighttime home blood pressure (BP) measured by a wrist‐type BP monitoring device. Forty‐six hypertensive patients (mean 69.0±11.6 years, 56.5% male) self‐measured their nighttime BP hourly using simultaneously worn wrist‐type and upper arm‐type nocturnal home BP monitoring devices at home on two consecutive nights. Using the average 7.4±1.3 measurements on the first night and the average 7.0 ± 1.8 measurements on the second night, the authors assessed the reliability and the reproducibility of nighttime BP measured on the two nights. The difference between nights in systolic BP (SBP) measured by the wrist‐device was not significant (1.6±7.0 mmHg, *p* = .124), while the difference in diastolic BP (DBP) was marginally significant (1.4±4.9 mmHg, *p* = .050). The intraclass correlation coefficients (ICCs) for agreement between nights were high both in SBP and DBP average (SBP: 0.835, DBP: 0.804). Averaging only three points of SBP resulted in lower ICC values, but still indicated good correlations (ICC > 0.6). On the other hand, the correlations of the standard deviation and average real variability of SBP between nights were low, with ICCs of 0.220 and 0.436, respectively. In conclusion, the average SBP values measured on the first night were reliable even when averaging only three readings. The reproducibility of nighttime BP variability seemed inferior to that of BP average; it might be better to measure nighttime BP over multiple nights to assess BP variability. However, this hypothesis needs verification in other study population. In addition, our study population had well‐controlled BP, which limits the generalizability of this findings to all hypertensive patients.

## 1 INTRODUCTION

Home blood pressure (BP) monitoring has been well recognized as an easy and reliable method for management of hypertension in clinical practice and is recommended by several international guidelines.^1^, ^2^, ^3^ Despite the popularity of home BP monitoring (HBPM), an optimal method to evaluate conventional home BP values (ie, BPs measured in the morning and/or evening) has not been fully established. The European Society of Hypertension recommends discarding BP values measured on the first monitoring day because the BP values obtained at first measurement are likely to be higher and more unstable compared to those obtained at subsequent measurements. However, the results from the Ohasama Study and the Finn‐Home Study showed that discarding the first day of measurements did not enhance the predictive ability.^4^, ^5^, ^6^

There is much less evidence and no consensus on nighttime home BP measurement, which can be measured by using a recently developed HBPM device with a function for automatic BP measurement during sleep. The Condition Study using an upper arm‐type nocturnal HBPM device demonstrated that the reproducibility of upper arm‐measured nighttime home BP values measured on the first night and the second night was high.^7^

A newly developed wrist‐type nocturnal HBPM device (HEM‐9601T; Omron Healthcare, Kyoto, Japan) reduces the discomfort caused by cuff inflation and measurement noise. The BP values measured by the HEM‐9601T were confirmed to be reliable both under laboratory conditions^8^ and sleeping conditions at home.^9^ In the present study, we investigated the reproducibility of nighttime home BP values measured by the wrist‐type HBPM device under real‐world sleeping conditions at home.

## 2 SUBJECTS AND METHODS

### 2.1 Study design

This was a post hoc analysis of data obtained from our comparison study of nighttime home BP readings measured by an HEM‐9601T wrist‐type device (NightView; Omron Healthcare) and an HEM‐9700T upper arm‐type device (Omron Healthcare).^9^ Details of the comparison study design have been published previously.^9^ Briefly, 50 hypertensive patients with antihypertensive treatment (mean age 68.9±11.3 years) were asked to measure their nighttime BP during sleep at home for two consecutive nights. They wore both the wrist and upper arm‐devices on the same non‐dominant arm and started the nighttime measurement mode just before going to bed each night.

During the nighttime measurement mode, the wrist device was preset to measure BP every hour on the hour (The measurement timing was changed for this study. The default setting is to measure at 2:00 am, 4:00 am, and 4 h after starting nighttime measurement mode.), and the upper arm‐device was preset to activate 3 min after the activation of the wrist device. Since the HEM‐9601T wrist device detects BP level during cuff inflation, it can avoid over‐pressurize and measurement noise, resulting in less sleep disturbance. The HEM‐9700T upper arm‐device detects BP level during cuff deflation. To reduce the influence of sequent measurement, especially due to sleep disturbance caused by cuff's inflation, the measurement protocol was set up in which the wrist device was activated first, followed by the upper arm‐device.

The study protocol was approved by the institutional review board of Jichi Medical University School of Medicine (rin‐A19‐241) and registered on a clinical trials registration site (University Hospital Medical Information Network Clinical Trials Registry, UMIN000041540). All participants provided written informed consent.

In the present reproducibility analysis, we used data obtained from 46 participants who successfully measured their nighttime home BP over two nights (four participants failed to measure their nighttime BP on one of the nights).

### 2.2 BP measurement devices

The HEM‐9601T (NightView) is an automatic oscillometric device for self‐measuring BP at the wrist, equipped with an algorithm for supine‐position measurements.^8^ The BP parameters measured by this wrist device was optimized to the parameters measured by a mercury sphygmomanometer at the upper arm both in the sitting and supine position. The HEM‐9700T is an automatic upper arm‐type device for the self‐measurement of BP.^10^ Both the wrist device and the upper arm‐device have an automatic BP measurement function during sleep.

### 2.3 Nighttime BP measurement index

The participants' nighttime BP values were measured every hour on the hour after going to bed during the nighttime measurement mode by both the HEM‐9601T and the HEM‐9700T with 3 min intervals between the two devices (HEM‐9601T first, then HEM‐9700T). Of those values, the average of three readings measured at 2:00, 3:00, and 4:00 a.m. (2:03, 3:03, and 4:03 a.m. for the HEM‐9700T measurements) was defined as a clock‐based index, and the average of three readings of the second, third, and fourth hourly measurements taken after the participant's bedtime (actually 61–120 min, 121–180 min, and 181–240 min after bedtime) was defined as a bedtime‐based index.

The variability of nighttime BP was assessed by calculating the standard deviation (SD) and average real variability (ARV). The SD reflects the dispersion of BP measurements without accounting for the order of BP measurements. The ARV averages the absolute differences of consecutive measurements.^11^

### 2.4 Statistical analyses

All statistical analyses were performed using the SAS ver. 9.4 software program (SAS Institute, Cary, NC, USA). The reproducibility of nighttime BP indexes between nights or between devices was assessed by testing for bias, association, and agreement.^12^ Differences between the nighttime BP indexes were tested using a paired *t*‐test. The intraclass correlation coefficient (ICC) for agreement using a two‐way random model of absolute agreement, that is, the ICC (2,1), was calculated to assess the association of nighttime BP values between devices or between nights. Bland–Altman plots were created to represent the mean bias and limits of agreement. Values of *p* < .05 were considered significant. All data processing and analyses were independently conducted at the Global Analysis Center of BP (GAP) at the Jichi Medical University COE Cardiovascular Research and Development (JCARD) Center.

## 3 RESULTS

### 3.1 Participant characteristics

All 50 participants in the comparison study were hypertensive patients receiving antihypertensive treatment and details of their characteristics were previously reported.^9^

Of the 50 patients, 46 patients were included in this reproducibility analysis. The mean age of the 46 patients was 69.0±11.6 years, 56.5% were male, and the average body mass index was 25.6±3.4 kg/m^2^. The prevalence of regular alcohol use was 26.1%; current smoking, 2.2%; diabetes mellitus, 19.6%; hyperlipidemia, 43.5%; chronic kidney disease, 4.4%; hyperuricemia, 23.9%; sleep apnea syndrome (SAS), 17.4%; history of cardiovascular disease, 10.9%; and history of heart failure, 4.4%.

### 3.2 Nighttime BP measured by the wrist HBPM device and the upper arm HBPM device

As previously reported, the self‐monitored nighttime home systolic BP (SBP) values measured by the wrist HBPM device equipped with a supine‐position algorithm were comparable to those measured by the upper arm HBPM device.^9^, ^13^ In a previous paper, we reasoned that the significantly lower diastolic BP (DBP) measured with a wrist device compared to that measured with an upper arm‐device could be due to the different BP algorithms used in the two devices.^9^ Among all the 50 participants, the ICCs for the agreement of each reading simultaneously taken by the wrist and upper arm HBPM device in SBP, DBP, and heart rate were 0.802, 0.686, and 0.877, respectively (Supplementary Table). The ICCs for the agreement of individual average values between devices were also high (SBP: 0.835–0.920; DBP: 0.694–0.796; heart rate: 0.831–0.908).

### 3.3 Nighttime average BP measured on the first night and the second night

Among the 50 participants, 46 participants successfully measured their nighttime BP on each of the two nights and were included in the analysis for the reproducibility of individual average BPs measured at night. Forty‐three participants measured BP at least once at 2:00, 3:00, or 4:00 on each night and were included in the analysis for the reproducibility of individual clock‐based average index. Forty‐five participants measured BP at least once at the second, third, or fourth hourly measurement points after going to bed on each night and were included in the analysis for the reproducibility of individual bedtime‐based average index.

Table 1 shows individual average parameters of nighttime BP for the first night and the second night. There was no significant difference between nights in the wrist‐measured SBP parameter when averaging all readings (1.6±7.0 mmHg, *p* = .124), three clock‐based readings (0.1±10.9 mmHg, *p* = .956), and three bedtime‐based readings (0.1±9.7 mmHg, *p* = .927). The ICCs (95% CI) for the agreement of these three SBP average indexes between nights were 0.835 (0.721–0.905), 0.690 (0.492–0.819), and 0.774 (0.622–0.869), respectively. The DBP average of all readings was lower on the second night (1.4±4.9 mmHg lower, *p* = .050), while their correlation was strong with an ICC of 0.804 (0.668–0.887). The heart rate average of three bedtime‐based readings was significantly higher on the second night (1.5±4.8 mmHg higher, *p* = .038), while the correlation of the readings was strong with an ICC of 0.740 (0.568–0.850). The other DBP and heart rate indexes showed no significant difference between nights and high ICC values. As for the BPs measured by the upper arm‐type device, all average parameters were comparable between nights. The ICCs of these average values were high and similar to the results for the wrist‐type device. The limits of agreement between the first and the second night are shown in Figure S1. The Bland–Altman plot indicated good agreement between nights in the averages of all readings of SBP, DBP, and heart rate measured by both devices.

**TABLE 1:** Comparison of nighttime home BP average parameters measured by the wrist‐type and upper arm‐type device between the first and the second nights

	Wrist‐type device					Upper arm‐type device				
	First night	Second night	Difference	*p* value^a^	ICC (2,1) [95% CI]	First night	Second night	Difference	*p* value^a^	ICC (2,1) [95% CI]
Individual averages of all readings per night (*N* = 46)										
Number of measurements	7.4±1.3	7.0±1.8	0.4±1.8	.157		7.4±1.3	7.0±1.8	0.4±1.8	.157	
SBP (mmHg)	117.0±11.8	115.4±12.8	1.6±7.0	.124	0.835 [0.721–0.905]	116.7±12.0	115.6±13.7	1.2±6.5	.232	0.872 [0.781–0.927]
DBP (mmHg)	68.0±7.4	66.5±8.5	1.4±4.9	.050	0.804 [0.668–0.887]	71.7±8.5	70.8±8.2	0.9±4.6	.213	0.850 [0.746–0.914]
Heart rate (bpm)	60.4±6.6	61.4±6.7	−0.9±4.4	.158	0.776 [0.630–0.870]	60.2±6.5	61.4±6.8	−1.2±4.5	.069	0.758 [0.601–0.859]
Individual averages of three clock‐based readings measured at 2:00, 3:00, and 4:00 a.m. per night (*N* = 43)										
Number of measurements	2.8±0.5	2.7±0.5	0.05±0.7	0.643		2.8±0.5	2.7±0.5	0.05±0.7	0.643	
SBP (mmHg)	115.2±13.1	115.2±14.3	0.1±10.9	0.956	0.690 [0.492–0.819]	116.4±15.0	115.8±15.6	0.7±11.6	0.715	0.716 [0.532–0.836]
DBP (mmHg)	67.2±7.5	66.0±8.3	1.2±7.0	0.282	0.605 [0.378–0.764]	71.1±10.0	71.2±9.3	−0.2±7.6	0.889	0.692 [0.496–0.821]
Heart rate (bpm)	60.0±7.1	60.0±6.6	0.02±6.0	0.987	0.615 [0.387–0.772]	59.8±7.1	60.2±6.2	−0.4±6.0	0.695	0.603 [0.370–0.764]
Individual averages of three bedtime‐based readings measured at the second, third, and fourth hourly measurement points after going to bed per night (*N* = 45)										
Number of measurements	3.0	2.96±0.3	0.04±0.3	0.323		3.0	2.96±0.3	0.04±0.3	0.323	
SBP (mmHg)	112.9±14.1	112.7±14.4	0.1±9.7	0.927	0.774 [0.622–0.869]	114.3±13.2	112.9±15.1	1.4±10.0	0.354	0.753 [0.593–0.856]
DBP (mmHg)	65.1±8.8	64.9±10.1	0.2±7.6	0.856	0.682 [0.486–0.812]	69.8±9.3	68.8±9.2	1.0±7.4	0.371	0.684 [0.492–0.812]
Heart rate (bpm)	60.7±6.6	62.2±7.2	−1.5±4.8	0.038	0.740 [0.568–0.850]	60.9±6.5	61.6±7.4	−0.7±5.2	0.353	0.724 [0.550–0.838]

Values are mean ± SD. ICC (2,1): two‐way random model of absolute agreement, single rating.

Difference: value on the first night minus that on the second night.

*Abbreviations*: BP, blood pressure; DBP, diastolic blood pressure; ICC, intraclass correlation coefficient; SBP, systolic blood pressure.

^a^ Paired *t*‐test for the difference between nights.

### 3.4 Variability of nighttime BP measured on the first night and the second night

Table 2 shows the averages of nighttime BP variability parameters for the first night and the second night. There was no significant difference between nights in the SD and ARV of SBP, DBP, and heart rate measured by both devices, except for the ARV of DBP measured by the upper‐arm device. The limits of agreement between the first and the second night were shown in the supplementary figures (Figure S2: SD values, Figure S3: ARV values). The Bland–Altman plot indicated good agreement between nights in all the SD and ARV indexes. However, the correlation of SD and ARV was poor with low ICC values both in the wrist‐type device (SBP: 0.220 for SD, 0.436 for ARV; DBP: −0.030 for SD, 0.047 for ARV; heart rate: 0.419 for SD, 0.516 for ARV) and the upper‐arm device (SBP: 0.205 for SD, 0.259 for ARV; DBP: 0.116 for SD, 0.250 for ARV; heart rate: 0.419 for SD, 0.131 for ARV).

**TABLE 2:** Comparison of nighttime home BP variability parameters measured by the wrist‐type and upper arm‐type device between the first and the second nights

	Wrist‐type device					Upper arm‐type device				
	First night	Second night	Difference	*p* value^a^	ICC (2,1) [95% CI]	First night	Second night	Difference	*p* value^a^	ICC (2,1) [95% CI]
Systolic BP										
SD of readings per night (mmHg)	11.2±4.7	10.0±3.4	1.1±5.1	.151	0.220 [−0.064–0.474]	9.7±4.5	10.2±4.0	−0.4±5.4	.579	0.205 [−0.092–0.467]
ARV of readings per night (mmHg)	10.9±5.5	11.2±3.9	−0.3±5.0	.683	0.436 [0.167–0.644]	9.2±4.8	10.5±3.9	−1.1±5.3	.178	0.259 [−0.024–0.506]
Diastolic BP										
SD of readings per night (mmHg)	7.7±3.6	7.3±2.8	0.5±4.6	.504	−0.030 [−0.319–0.263]	6.6±3.0	7.1±3.6	−0.5±4.4	.452	0.116 [−0.180–0.392]
ARV of readings per night (mmHg)	7.3±4.2	7.7±2.6	−0.3±4.8	.666	0.047 [−0.250–0.333]	6.2±3.2	7.3±2.8	−1.2±3.7	.042	0.250 [−0.024–0.495]
Heart rate										
SD of readings per night (bpm)	4.1±2.1	4.1±2.3	−0.02±2.4	.967	0.419 [0.146–0.632]	4.1±1.9	4.1±2.1	0.01±2.2	.969	0.419 [0.146–0.632]
ARV of readings per night (bpm)	3.8±2.2	4.3±1.9	−0.5±2.2	.167	0.516 [0.273–0.699]	3.8±2.0	4.2±2.1	−0.3±2.8	.426	0.131 [−0.165–0.405]

Values are mean ± SD. ICC (2,1): two‐way random model of absolute agreement, single rating.

Difference: value on the first night minus that on the second night.

*Abbreviations*: ARV, average real variability; BP, blood pressure; ICC, intraclass correlation coefficient; SD, standard deviation.

^a^ Paired *t*‐test for the difference between nights.

Among the 38 patients who had not been previously diagnosed with SAS, the ICCs for the SD and ARV of SBP were slightly improved (wrist‐device: 0.278 for SD and 0.465 for ARV; upper arm‐device: 0.290 for SD and 0.383 for ARV).

## 4 DISCUSSION

This is the first study to investigate the reproducibility of nighttime home BP measured by a wrist‐type HBPM device. The results indicate that the reproducibility of nighttime BP variability was inferior to that of BP average.

### 4.1 The first day measurement

In the present study, the average of the wrist‐measured SBP on the first night was comparable to that on the second night, whether averaged over all 60‐min interval measurements, averaged over three time points at 2, 3, and 4 o'clock, or averaged over three readings measured at the second, third, and fourth hourly measurement points after bedtime. Regarding home daytime BP measurements (ie, morning and evening measurements), some studies have compared morning and/or evening home BP measured on the first day and the second day. Stergiou and colleagues concluded that the first day gives higher and unstable BP values.^14^ A Japanese study also demonstrated that both morning and evening home BP measured on the first day were significantly higher than those on the second day.^15^ However, fewer reports have been published on the reliability of nighttime home BP measured on the first day. Fujiwara and colleagues demonstrated that there was no difference in the nighttime home BP measured by an upper arm nocturnal HBPM device between the first and the second night.^7^ Consistent with the results of their study, our present results showed that both the wrist‐measured and upper arm‐measured nighttime BP levels measured on the first day were comparable to those measured on the second day. Thus, the first night BP readings measured by either the wrist‐type or upper arm‐type HBPM were reliable and could be included in the evaluation. Nighttime home BP measured using an automatic function is less likely to be affected by psychological factors. This might be partly responsible for the fact that the nighttime home BP measured on the first day was not higher than that measured on later days.

### 4.2 The reproducibility of wrist‐measured nighttime BP average

The correlation of wrist‐measured overnight BP average between the first and the second night was fairly good. Recent studies using a home nocturnal BP monitoring device have employed three preset measurements per night at 2, 3, and 4 a.m. or at 2, 3, and 4 h after bedtime.^16^ When the three time points of BP readings were averaged based on clock time or bedtime, the ICC values were lower compared with that of the overnight average but still indicated good correlation both in the wrist and upper arm measurements. The Bland–Altman plot showed good agreement between nights in the averages of all readings of SBP and DBP measured by both devices.

In the results of paired *t*‐test, there was no significant difference between nights in the SBP average indexes both in the wrist and upper‐arm measurements, while the wrist‐measured DBP average of all readings showed marginally significant differences between nights (1.4±4.9 mmHg, *p* = .050). However, the Bland–Altman plot did not show any notable difference and its ICC value was high (0.804). In addition, no significant difference in DBP average of three time points (ie, clock‐based average and bed‐time based average) was observed. Therefore, we considered this difference is not clinically important.

Based on these results of the correlation, agreement, and bias, the reproducibility of the wrist‐measured nighttime SBP average between nights was good, even when only three measurements were taken per night. However, the reproducibility of the wrist‐measured nighttime DBP average needs further verification.

### 4.3 The reproducibility of wrist‐measured nighttime BP variability

In this study, the correlation of SD assessed by ICC was poor both in the wrist‐measured BP and the upper arm‐measured BP, but that of ARV was better than SD. Our results were consistent with a previous study that assessed the reproducibility of BP variability measured by ABPM.^17^ The ARV is considered to be more representative of time series variability than SD, and it has been reported that ARV adds prognostic value to the ABPM readings.^11^

A previous study that evaluated the reproducibility of nighttime BP in patients suspected of having obstructive sleep apnea demonstrated that SAS causes poor reproducibility of the SD of nighttime SBP measured at a fixed‐interval.^18^ In the present study, the ICC value of the SBP variability index was improved by excluding the eight patients with SAS from the analysis.

As nighttime BP variability is an important risk factor for cardiovascular risk,^19^ the assessment for nighttime BP variability is essential for hypertension treatment. From these results, which showed good reproducibility of SBP average but not good reproducibility of SBP variability indexes, we hypothesized that measuring nighttime BP over multiple days is needed to evaluate cardiovascular risk, even for patients without SAS.

### 4.4 Study limitations

There are several possible limitations to our study. First, the sample size for this reproducibility analysis was small (*n* = 46), and we assessed only two nights of BP readings. In addition, our study patients had well‐controlled nighttime BP and it is unclear whether the results in this study can be applied to uncontrolled hypertensive patients. Second, we assessed the reproducibility by testing for bias, association, and agreement. Inconsistent results between mean difference and ICC were observed in variability indexes. The ICC has a limitation that it resides in its dependence on the variance of the assessed population.^20^ That is, the ICCs for BP averages may be overestimated and the ICCs for variability indexes may be underestimated. Therefore, the results of this study are needed to be interpreted carefully. Further prognostic studies with longer nighttime measurement schedule in larger populations are needed to confirm the reproducibility of nighttime BP for wrist BP monitoring.

## 5 CONCLUSIONS AND PERSPECTIVES

In the 46 hypertensive patients, the average SBP values measured on the first night were reliable even when averaging only three readings. The reproducibility of wrist‐measured nighttime SBP average between the first and second nights was good, whereas the reproducibilities of SD and ARV seem to be inferior to that of BP average. These results indicate that it might be better to repeatedly measure nighttime BP over multiple nights to assess BP variability. However, this hypothesis needs verification in other study populations including uncontrolled hypertensive patients.

## CONFLICT OF INTEREST

K. Kario has received research grants from Omron Healthcare, A&D, and Fukuda Denshi.

## AUTHOR CONTRIBUTIONS

Kazuomi Kario supervised the conduct of the study and data analysis, and had the primary responsibility of writing this paper. Naoko Tomitani analyzed the data and wrote the Introduction, Methods, Results, and Discussion sections. Hiroshi Kanegae contributed to the analysis. All authors discussed the results and contributed to the final manuscript.

## Supporting information

Supplementary information
