# Supplementary material for: Reproducibility of nighttime home blood pressure measured by a wrist‐type nocturnal home blood pressure monitoring device
Source: J Clin Hypertens (Greenwich). 2021 Aug 21;23(10):1872–8. doi: 10.1111/jch.14342 (PMC8678720; doi:10.1111/jch.14342)
Supplement: Supplementary file 1 — Supplementary information [file JCH-23-1872-s001.docx]

***Supplementary Materials***

**Reproducibility of nighttime home blood pressure measured by a wrist-type**

**nocturnal home blood pressure monitoring device**

**Short title:** Reproducibility of wrist-measured nighttime HBP

Naoko Tomitani, BSc^1^, Hiroshi Kanegae, BSc^1,2^, Kazuomi Kario, MD, PhD^1,^*

^1^Division of Cardiovascular Medicine, Department of Medicine, Jichi Medical University School of Medicine, Tochigi, Japan

^2^Genki Plaza Medical Center for Health Care, Tokyo, Japan

***Corresponding author:** Kazuomi Kario, MD, PhD

**Supplementary Table.** Comparison of BP parameters measured by the wrist-type and upper-arm-type device in the 2-night home nocturnal setting (total 694 pairs of measurements from 50 patients)

|  | Wrist device | Upper arm device | Difference  (Wrist – Upper arm) | P for difference* | ICC (2,1)  [95% CI] |
| --- | --- | --- | --- | --- | --- |
| All measurements (n=694 pairs of measurements) | | |  |  |  |
| SBP, mmHg | 116.4±16.2 | 116.2±16.3 | 0.2±10.2 | 0.563 | 0.802 [0.774 – 0.827] |
| DBP, mmHg | 66.8±10.8 | 70.9±10.9 | -4.1±7.9 | <0.001 | 0.686 [0.513 – 0.786] |
| Heart rate, bpm | 60.6±7.9 | 60.5±7.8 | 0.1±3.9 | 0.571 | 0.877 [0.859 – 0.893] |
| Individual averages of all readings per night  (n=96 pairs of average BP [mean 7.2±1.5 measurements/night] ) | | | |  |  |
| SBP, mmHg | 116.5±12.2 | 116.3±12.7 | 0.2±5.4 | 0.764 | 0.920 [0.862 – 0.954] |
| DBP, mmHg | 67.2±8.0 | 71.2±8.3 | -4.0±4.4 | <0.001 | 0.796 [0.210 – 0.925] |
| Heart rate, bpm | 60.8±6.6 | 60.7±6.6 | 0.1±1.7 | 0.624 | 0.908 [0.843 – 0.947] |
| Individual averages of three readings measured at 2:00, 3:00, and 4:00 a.m. per night  (n=93 pairs of average BP [mean 2.8±0.5 measurements/night]) | | | |  |  |
| SBP, mmHg | 115.8±13.4 | 116.4±14.9 | -0.6±7.6 | 0.420 | 0.835 [0.726 – 0.902] |
| DBP, mmHg | 66.8±8.0 | 71.3±9.4 | -4.4±5.8 | <0.001 | 0.694 [0.233 – 0.862] |
| Heart rate, bpm | 59.9±6.7 | 59.9±6.6 | 0.1±2.6 | 0.838 | 0.831 [0.719 – 0.900] |
| Individual averages of three readings measured at 2, 3, and 4 h after going to bed  (n=93 pairs of average BP [mean 3 measurements/night]) | | | |  |  |
| SBP, mmHg | 113.1 ± 14.1 | 113.6 ± 13.9 | -0.6±6.3 | 0.424 | 0.878 [0.795 – 0.929] |
| DBP, mmHg | 65.0 ± 9.4 | 69.3 ± 9.4 | -4.3±5.4 | <0.001 | 0.748 [0.265 – 0.894] |
| Heart rate, bpm | 61.4 ± 6.9 | 61.2 ± 6.9 | 0.2±2.6 | 0.399 | 0.883 [0.803 – 0.932] |

Data are partly adapted from previous reports [9, 13].

Values are mean ± SD. ICC(2, 1): two-way random model of absolute agreement, single rating.

Difference: value on the 1st night minus that on the 2nd night.

BP, blood pressure; SBP, systolic blood pressure; DBP, diastolic blood pressure; ICC, intraclass correlation coefficient.

*Paired t-test for the difference between nights.* paird t-test for the difference between nights

**Supplementary Figure S1.** Bland-Altman plots of the differences between the average values of nighttime blood pressure and heart rate measured on the first and the second night.


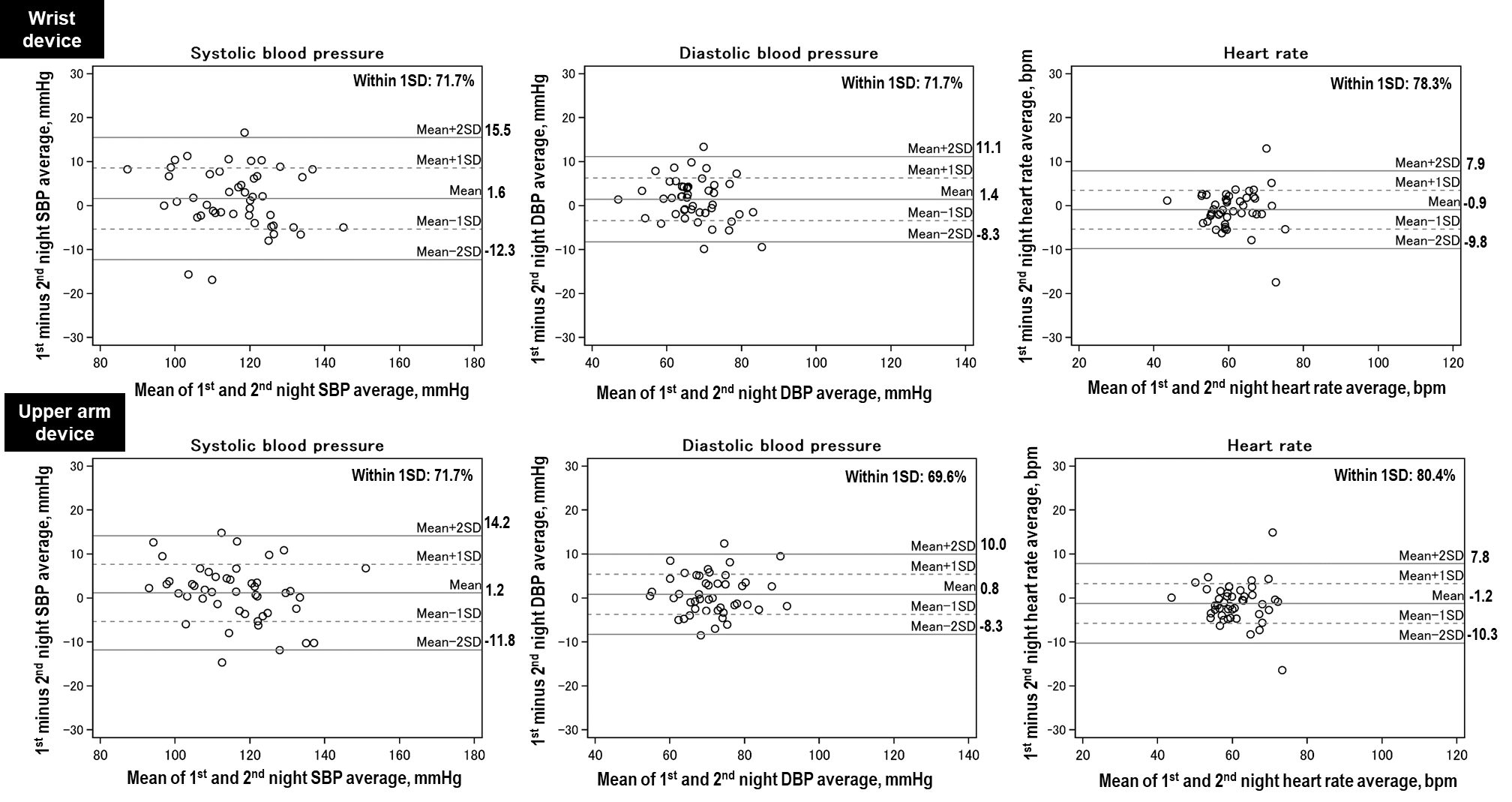


**Upper:** Plots of average values measured by the wrist device. **Lower:** Plots of averaged values measure by the upper arm device.

Solid lines represent mean and mean±2SD. Dotted lines represent mean±1SD.

**Supplementary Figure S2.** Bland-Altman plots of the differences between the SD values of nighttime blood pressure and heart rate measured on the first and the second night.


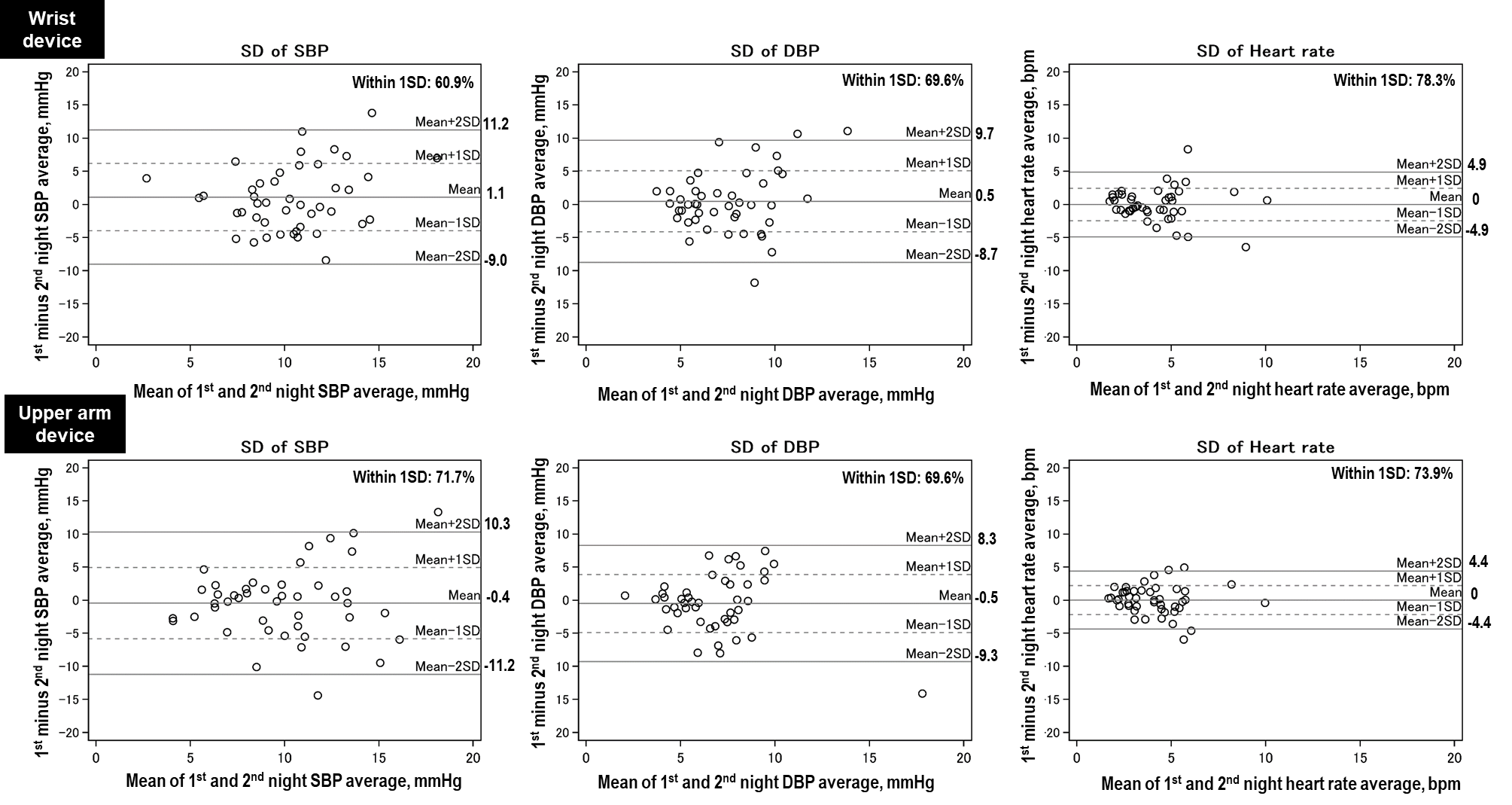


**Upper:** Plots of average values measured by the wrist device. **Lower:** Plots of averaged values measure by the upper arm device.

Solid lines represent mean and mean±2SD. Dotted lines represent mean±1SD.

**Supplementary Figure S3.** Bland-Altman plots of the differences between the ARV values of nighttime blood pressure and heart rate measured on the first and the second night.


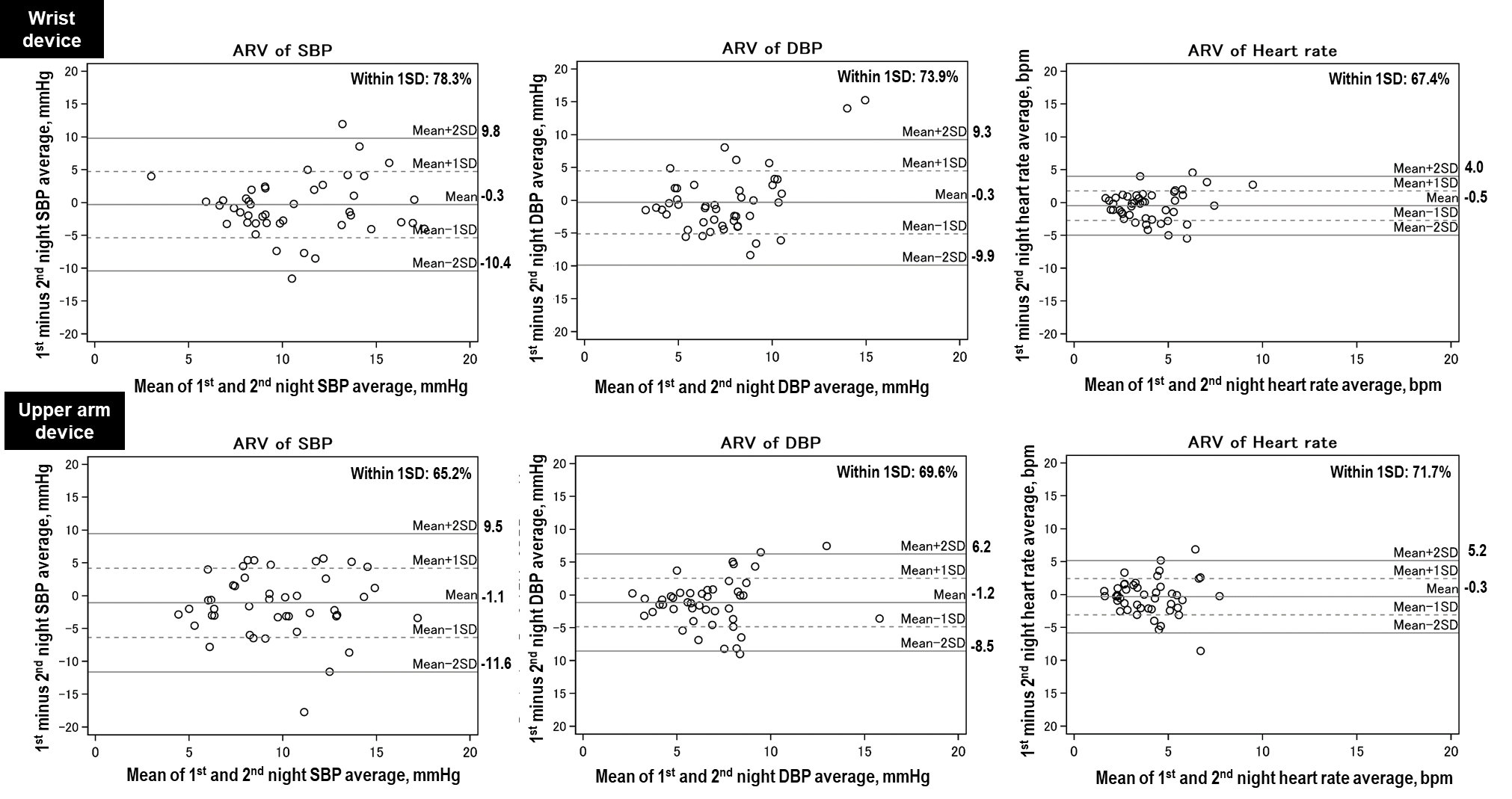


**Upper:** Plots of average values measured by the wrist device. **Lower:** Plots of averaged values measure by the upper arm device.

Solid lines represent mean and mean±2SD. Dotted lines represent mean±1SD.
